# Clinical and molecular consequences of disease-associated de novo mutations in *SATB2*

**DOI:** 10.1038/gim.2016.211

**Published:** 2017-02-02

**Authors:** Hemant Bengani, Mark Handley, Mohsan Alvi, Rita Ibitoye, Melissa Lees, Sally Ann Lynch, Wayne Lam, Madeleine Fannemel, Ann Nordgren, H. Malmgren, M. Kvarnung, Sarju Mehta, Shane McKee, Margo Whiteford, Fiona Stewart, Fiona Connell, Jill Clayton-Smith, Sahar Mansour, Shehla Mohammed, Alan Fryer, Jenny Morton, UK10K Consortium, Detelina Grozeva, Tara Asam, David Moore, Alejandro Sifrim, Jeremy McRae, Matthew E. Hurles, Helen V. Firth, F. Lucy Raymond, Usha Kini, Christoffer Nellåker, DDD study, David R. FitzPatrick

**Affiliations:** 1 MRC Human Genetics Unit, IGMM, University of Edinburgh, Western General Hospital, Edinburgh, UK; 2 Avdeling for Medisinsk Genetikk, Oslo Universitetssykehus, Oslo, Norway; 3 Department of Clinical Genetics, Oxford University Hospitals NHS Trust, Oxford, UK; 4 North East Regional Genetics Service, Great Ormond Street Hospital, London, UK; 5 National Centre for Medical Genetics, Our Lady’s Children’s Hospital, Dublin, Ireland; 6 South East Scotland Genetic Service, Western General Hospital, Edinburgh, UK; 7 Clinical Genetics Unit, Department of Molecular Medicine and Surgery, Karolinska Institutet, Stockholm, Sweden; 8 Department of Clinical Genetics, Cambridge University Hospitals NHS Foundation, Cambridge, UK; 9 Northern Ireland Regional Genetics Centre, Belfast City Hospital, Belfast, UK; 10 West of Scotland Genetic Services, Queen Elizabeth University Hospital, Glasgow, UK; 11 South East Thames Regional Genetics Service, Guy’s and St Thomas’ NHS Foundation Trust, London, UK; 12 Genetic Medicine, North West Regional Genetics Service, Manchester, UK; 13 Department of Clinical Genetics, St Georges Hospital, Tooting, UK; 14 Cheshire & Merseyside Regional Genetics Service, Liverpool Women’s NHS foundation Trust, Liverpool, UK; 15 West Midlands Regional Genetics Service, Birmingham Women’s NHS Foundation Trust, Birmingham, UK; 16 Wellcome Trust Sanger Institute, Wellcome Genome Campus, Cambridge, UK; 17 Cambridge Institute for Medical Research, University of Cambridge, Cambridge, UK; 18 South-East Scotland Regional Genetics Laboratories, Western General Hospital, Edinburgh, UK; 19 Spires Cleft Centre, John Radcliffe Hospital, Oxford, UK; 20 Nuffield Department of Obstetrics & Gynaecology, University of Oxford, Women’s Centre, John Radcliffe Hospital, Oxford, UK; 21 Department of Engineering Science, University of Oxford, Institute of Biomedical Engineering, Oxford, UK; 22 Big Data Institute, University of Oxford, Oxford, UK

**Keywords:** absent speech, CUT domain, de novo mutation, intellectual disability, SATB2

## Abstract

**Purpose:**

To characterize features associated with de novo mutations affecting *SATB2* function in individuals ascertained on the basis of intellectual disability.

**Methods:**

Twenty previously unreported individuals with 19 different *SATB2* mutations (11 loss-of-function and 8 missense variants) were studied. Fibroblasts were used to measure mutant protein production. Subcellular localization and mobility of wild-type and mutant SATB2 were assessed using fluorescently tagged protein.

**Results:**

Recurrent clinical features included neurodevelopmental impairment (19/19), absent/near absent speech (16/19), normal somatic growth (17/19), cleft palate (9/19), drooling (12/19), and dental anomalies (8/19). Six of eight missense variants clustered in the first CUT domain. Sibling recurrence due to gonadal mosaicism was seen in one family. A nonsense mutation in the last exon resulted in production of a truncated protein retaining all three DNA-binding domains. SATB2 nuclear mobility was mutation-dependent; p.Arg389Cys in CUT1 increased mobility and both p.Gly515Ser in CUT2 and p.Gln566Lys between CUT2 and HOX reduced mobility. The clinical features in individuals with missense variants were indistinguishable from those with loss of function.

**Conclusion:**

*SATB2* haploinsufficiency is a common cause of syndromic intellectual disability. When mutant SATB2 protein is produced, the protein appears functionally inactive with a disrupted pattern of chromatin or matrix association.

*Genet Med* advance online publication 02 February 2017

## Background

*SATB2* (special AT-rich sequence-binding protein 2) was originally identified as a gene disrupted by breakpoints in two different de novo apparently balanced chromosomal rearrangements involving distal 2q32 in unrelated girls with cleft palate, a distinctive facial appearance, and intellectual disability.^1,2^ It is now recognized that de novo heterozygous single-nucleotide variants in *SATB2* are one of the most common causes of syndromic ID, accounting for ~0.3% of all analyzed affected individuals in the Deciphering Developmental Disorders (DDD) study.^3^ The distinct site-specific and stage-specific expression pattern of *Satb2* in embryonic mouse palatal shelves suggested a direct role in the etiology of both cleft palate and ID. This was subsequently confirmed by targeted inactivation of *Satb2* in mice, which demonstrated a dosage-sensitive role for the gene product in midline craniofacial patterning, osteoblast differentiation,^4^ and determining the fates of neuronal projections in the developing cerebral cortex.^5,6^

The first intragenic point mutation in *SATB2* was reported in 2007, by Leoyklang et al.,^7^ in a 36-year-old man with a heterozygous nonsense mutation in *SATB2* associated with a cleft palate, generalized osteoporosis, profound intellectual disability, epilepsy, and a jovial personality. Other de novo structural chromosomal anomalies, including *SATB2* disruptive breakpoints,^8,9^ intragenic deletions,^10^ and intragenic duplications,^11^ provided further evidence for an association with cleft palate and cognitive impairment. More recently, genome-wide sequencing studies have reported seven additional individuals with de novo heterozygous single-nucleotide variants in *SATB2* with phenotypes that overlap that previously associated with haploinsufficiency.^12,13,14^

The *SATB2* transcription unit spans 195.6 kb of genomic DNA (chr2:200,134,223-200,329,831 hg19) encoding a DNA-binding protein with a remarkably high level of conservation in primary sequence throughout vertebrate evolution.^15^ The gene lies telomeric to a gene desert containing multiple *cis*-regulatory elements essential for normal developmental expression of *SATB2*.^16^ CUT is a class of divergent homeodomain first identified in the cut protein encoded by the *Drosophila melanogaster* gene *ct*. SATB2 has two CUT domains (amino acids 352–437 and amino acids 482–560) and a classic homeodomain (amino acids 614–677).^2^

We report molecular and phenotypic features of 20 previously unreported individuals with heterozygous de novo probable loss-of-function mutations in *SATB2* identified by whole-exome sequencing (WES). Comparing the clinical features in these individuals with those from previously reported individuals has enabled further delineation of the *SATB2*-associated syndrome. The eight individuals with missense variants in this gene have clinical features indistinguishable from those with clear loss-of-function mutations. One of the families shows evidence for gonadal mosaicism as the cause of sibling recurrence of an apparently de novo variant in *SATB2*.

## Materials and Methods

### Subjects and clinical data

Sixteen of the 20 affected individuals included in this study were identified via trio-based WES as part of the DDD study (http://www.ddduk.org) Study. This study was approved by the UK Multicentre Research Ethics Committee under reference 10/H0305/83, and written consent was obtained from all participating families. Quantitative and categorical phenotypic data were extracted from the DDD patient information database. Non-DDD case information was supplied by the referring clinician. Two affected individuals were identified via WES analyses performed within separate clinical genetics laboratories; KAROL1 was referred to the Department of Clinical Genetics, Karolinska University Hospital, for dysmorphological and diagnostic evaluation. Written informed consent for inclusion in the study and consent for the publication of photographs were obtained from the legal guardians, with the study approved by the regional ethics committee in Stockholm. The remaining two individuals (29089 and 29090) were siblings in whom the same mutation was identified via a custom solution capture-targeted resequencing analysis of a large cohort with previously unexplained intellectual disability representing part of the GOLD study (Genetics of Learning Difficulties).

### WES and targeted resequencing

Details of the clinical ascertainment, WES strategy, variant calling, and filtering pipelines used in the DDD study are described elsewhere.^17^ Briefly, genomic DNA from affected individuals and their parents was extracted from blood or saliva and subjected to target capture using Agilent SureSelect 55MB Exome Plus and sequenced on Illumina HiSeq. De novo sequence variants were identified using DeNovoGear (https://github.com/denovogear) and the effect of each genomic variant was predicted with the Ensembl Variant Effect Predictor (grch37.ensembl.org/Homo_sapiens/Tools/VEP). For individual KAROL1, WES was performed in a family trio-based study using Illumina technology (Illumina, San Diego, CA). The sequencing was performed at Oxford Gene Technology and sequence data were returned and analyzed using software supplied by Oxford Gene Technology. Prior to the WES analysis array, comparative genomic hybridization was performed for all individuals with normal results. Details of the clinical ascertainment and targeted exome sequencing, variant calling, and filtering pipeline used in the GOLD study, a subset of the UK10K project, to identify siblings with mutations are described elsewhere.^18^ All mutations have been validated at least once as de novo using independent sequencing technologies (Sanger sequencing and/or targeted pull-down with next-generation sequencing).

### 3D modeling of mutations

3D modeling of structural effects was performed using the mutate_model script in MODELLER^19^ on available solution structures for residues 350–437 (RCDB PDB 1WIZ) for the CUT1 domain and 473–560 (RCDB PDB 2CSF) for the CUT2 domain. The resulting models were visualized using a SWISS-PdbViewer.^20^

### Average face analysis

To visualize the characteristic facial features of patients with shared mutations in the same gene, we generated realistic, de-identified average faces from unstandardized 2D facial photographs taken at clinic appointments. This was achieved by a fully automated, three-step algorithm that detects the location of a face within an image, identifies the locations of 36 facial feature points on the face, and creates composites from multiple faces using their respective facial feature points. Faces were detected using a discriminately learned deformable parts model detector.^21^ The resulting detection box was used to initialize the facial feature point annotation algorithm. We used the Supervised Descent Method approach detailed in ref. 22, which we trained using a data set of 3,100 manually annotated images.^23^ The resulting annotations were used to create a face mesh for each using Delaunay triangulation.

Our previously reported face averaging algorithm^23,24^ was improved on with the introduction of left–right facial asymmetry preservation and better de-identification of individuals. The averaging algorithm was initialized with a target face mesh, which was created by averaging the facial feature point constellations for 2,000 healthy individuals. The face mesh of each patient was aligned to this target mesh with respect to the points across the middle of the face (from forehead to chin). We then compared the Euclidean distances between each feature point pair on the patient’s mesh and the target. The relative difference between points on the right and left sides of the face was used as a measure of left–right facial asymmetry and faces were mirrored such that the side with greater deviance from the target mesh was on the same side for all patients. Once the asymmetries were taken into account, we created average face meshes for each group of patients that shared a mutation. The average face was created by morphing the image of each patient’s face onto this average face mesh. To avoid biases toward individuals with multiple images, we computed an average face for each patient and used these personal averages to compute the final average face. Finally, to avoid variances in illumination between images, which could cause any image in the composite to dominate, we normalized the pixel values within the face to an average value across all faces for each average.

### Plasmids, cell culture, and Photobleaching Experiments

Full-length human SATB2 cDNA was amplified from the human fetal brain cDNA library. The polymerase chain reaction product was cloned downstream of green fluorescence protein (GFP) in the Gateway pcDNA-DEST53 Vector according to the manufacturer’s protocol, resulting in N-terminal fusion protein. Site-directed mutagenesis was used to make mutant constructs. HeLa and human fibroblast cell lines were grown at 37 °C in 5% CO_2_ in Dulbecco’s modified Eagle’s medium containing 10% fetal bovine serum and 1% penicillin/streptomycin. Cells were seeded onto glass-bottom dishes (Mattek) and transfections were performed using Lipofectamine 2000 (Life Technologies) according to the manufacturer’s instructions. Up to 0.5 µg of each SATB2 construct was used per transfection. Media was substituted for Hank’s balanced salt solution 18–24 h after transfection and imaging was performed using a Nikon A1R confocal microscope equipped with the Nikon Perfect Focus System using a 60× oil immersion objective with a 1.4 numerical aperture. Using bleaching experiments, the pinhole was set to airy2 and digital zoom parameters were kept constant for each cell type. GFP was excited using a 488-nm laser, and emitted light was collected at 500–550 nm. Dishes of control and mutant cells were alternated over the course of imaging. Cells expressing constructs at comparable levels, with recorded fluorescence intensity within the dynamic range of the detector at similar gain settings, were selected for analysis. Data shown are representative of three independent experiments (*n* ≥ 21 cells per SATB2 construct).

### Cell lines, fractionation, western blotting, and antibodies

Stable cell lines were generated by Flp recombinase–mediated integration using HEK-293-Flp-In T-REx host cells (ThermoFisher) transfected with pcDNA5/FRT/TO-EGFP (EGFP-SATB2 or Mutant SATB2) and pCAGGS-Flp. Transfected cells were selected using 5 μg/ml blasticidin and 400 μg/ml hygromycin and protein expression was induced with 1 μg/ml tetracycline treatment. Subcellular fractions were prepared according to the method of de Seo et al.^25^ HEK-293-Flp-In T-REx expressing full-length or mutant EGFP-SATB2 were treated with 0.5% Triton X-100 in cytoskeleton buffer (10 mmol/l PIPES (piperazine-N,N’-bis(2-ethanesulfonic acid)), pH 6.8, 100 mmol/l NaCl, 300 mmol/l sucrose, 3 mmol/l MgCl_2_, and 1 mmol/l EGTA) supplemented with 1 mmol/l phenylmethanesulfonyl fluoride and 1× protease inhibitor mixture (Roche Applied Science) on ice. The supernatant was recovered after centrifugation and referred to as the soluble fraction. The insoluble fraction was suspended in cytoskeleton buffer and treated with 20 units of DNase I (Roche Applied Science) for 15 min at 37 °C; ammonium sulfate was added to a final concentration of 0.25 M. The soluble material was referred to as the chromatin fraction, and the insoluble fraction was washed with 2 M NaCl. The remainder (nuclear matrix fraction) was dissolved in 8 M urea and 10 mmol/l Tris-HCl, pH 8.0. The same proportion of each fraction was analyzed by western blot using anti-GFP antibody (sc-8334; Santa Cruz Biotechnologies, Dallas, TX). Anti-histone H3 (ab1791; Abcam, Cambridge, UK) and anti-lamin (sc-6216; Santa Cruz Biotechnologies) antibodies were used as chromatin and matrix fraction loading control. Immunoblotting was performed according to the standard protocol. Protein extracts from control and patient human fibroblast cell lines were mixed with 4× loading dye (Invitrogen), boiled, and separated on Novex 4–12% Bis-Tris Protein Gels (Invitrogen), followed by transfer onto polyvinylidene fluoride membranes. An affinity-purified antibody raised against an N-terminal His-tag fusion of the C-terminal half of human SATB2 (a.a. 329–733) was raised in rabbits as previously described.^16^ Enhanced chemiluminescence reagents (Amersham) were used for antibody detection. All cell lines were checked for mycoplasma contamination prior to use.

## Results

### Consequences of disease-associated de novo mutation in *SATB2*

**Figure 1:**
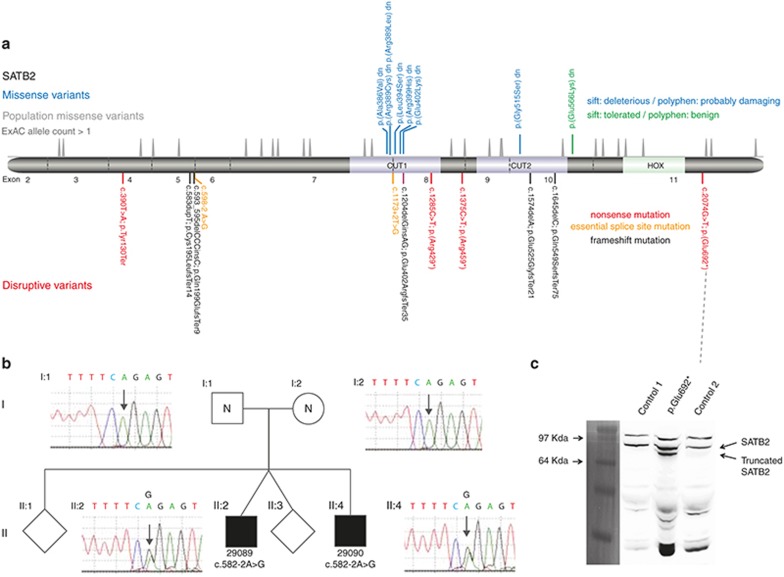
**SATB2 mutation spectrum and consequence.** (**a**) The relative positions of the de novo mutation identified in this article are indicated in a cartoon representation of the SATB2 wild-type protein, which includes the location of the DNA-binding domains. Below the protein diagram are the mutations that are predicted to result in disruption of the open reading frame. The mutations are color-coded by type: nonsense (red), frameshift (purple), and essential splice site (orange). The intron–exon boundaries of the gene encoding the protein are shown as vertical gray lines and the exon number given between these. Above are the missense variants, which have been color-coded to represent the predicted effect of the residue substitution. Immediately above the protein cartoon is a density plot representing the position of all missense variants seen more than once in the Exome Aggregation Consortium database. (**b**) The family structure and cognate chromatographs are shown of the family with apparent gonadal mosaicism. The affected boys (II:2 29089 and II:4 29090) share the same essential splice site mutation, which is absent in both parents (I:1 and I:2). (**c**) Photograph of a western blot of protein derived from cultured fibroblasts of two unrelated control individuals and case 14 with a nonsense mutation in the final exon using an antibody made for the C-terminal region of SATB2. A pre-stained protein ladder (Invitrogen LC5925) was used to assess the size of the bands. The same membrane was imaged using both white light and chemiluminescence and the cropped ladder image was size matched and aligned to the immunoblot. The expected SATB2 band of ~80 kDa is seen in all three, with a band of unknown identity above this. A lower band, consistent with the production of the 692-amino-acid C-terminally truncated version on SATB2, is seen in case 14 but not in the control.

Eleven of the 19 de novo *SATB2* variants reported here were predicted to be clearly disruptive to protein production, resulting in loss of function (**Figure 1a**, bottom panel). Two of 19 variants disrupted an essential splice site consensus sequence: one affecting the donor (5’) site at the end of exon 5 and one altering the acceptor (3’) site at the start of exon 8. Four of 19 were nonsense (stop gained) variants: one in exon 4, two in exon 8, and one in the last exon (exon 11). We identified 5/19 as frameshift variants, with each occurring 5’ to the last intron exon boundary of the gene (significant).

Eight of the variants predicted nonsynonymous changes in the open reading frame (**Figure 1a**, top panel). Seven of eight were located in the center of a CUT domain: six in CUT1 and one in CUT2. Each of the CUT domain variants had SIFT and PolyPhen scores that predicted a significant perturbation in protein function. The final missense variant (p.Glu566Lys) was in the region between the CUT2 and HOX domain. SIFT and PolyPhen predicted this variant to be tolerated or benign. Using Exome Aggregation Consortium data, we created density plots of the number of nonsynonymous variants that have been observed in the open reading frame in a population that was not selected for developmental disease (**Figure 1a**, middle panel). No variants were observed in the central portions of either CUT domain, suggesting the operation of purifying selection within the human population at these regions. There was evidence for constraint within the HOX domain, but the pattern was less striking on the density plot.

### Evidence for gonadal mosaicism in SATB2 de novo mutations

One of the essential splice variants (c.598-2 A>G) was identified as a heterozygous variant in two brothers (29089 and 29090) who had been recruited to the GOLD study with suspected X-linked intellectual disability. Subsequent analysis of the family revealed normal sequence at this base in peripheral blood–derived DNA from both parents. Biological relationships within the family were confirmed using a panel of highly informative single-nucleotide polymorphisms. These findings indicated that gonadal mosaicism was present in one of the parents. We were not able to determine the parental origin of the mutation because of the lack of informative markers on the parental alleles.

### Stop-gain in the last exon escapes nonsense-mediated decay

The stop-gain mutation in the last exon would be expected to escape nonsense-mediated decay. The predicted protein would have all three DNA-binding domains intact but would lack the final 41 amino acids. To determine whether this protein is produced, we established a fibroblast cell line from a skin biopsy derived from the affected individual. Western blot using protein extract from these fibroblasts and two control fibroblast lines showed the presence of the expected SATB2 band of ~80 kDa in all three cell extracts (**Figure 1c**). An additional, smaller band was present only in the patient-derived fibroblasts. The size of this band was consistent with the predicted 692aa protein product of the mutant cDNA. This supports the hypothesis that this mutant mRNA escapes nonsense-mediated decay.

### *SATB2* missense mutations alter the DNA-binding domain structure and nuclear mobility

**Figure 2:**
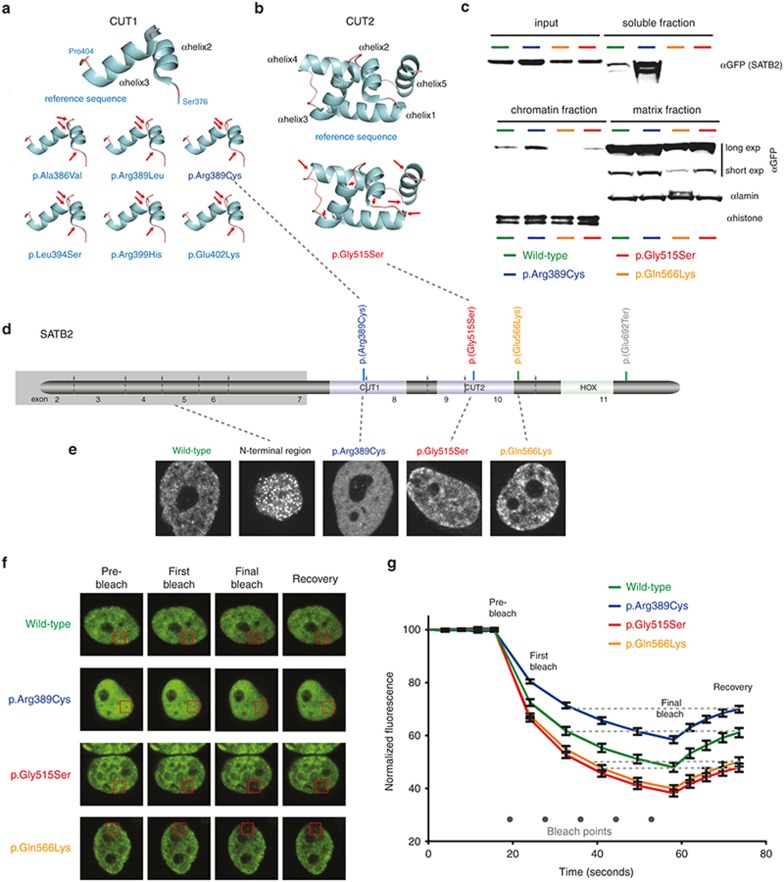
**Effect of mutation within DNA-binding domains of SATB2.** In silico predictions of the structural effects of missense mutations affecting CUT1 (**a**) or CUT2 (**b**) are shown. In each case, the boundaries of alpha helixes 3 and 2 have been altered (red arrows). The position of the cognate mutation within the DNA-binding domain is shown on a cartoon of the SATB2 protein in **d**. (**c**) Western blot analysis of fractionated HEK293 cells with Tetracyclin (TET) inducible expression of GFP-tagged mutant and wild-type SATB2. Most SATB2 is attached to the matrix. The CUT1 missense mutation results in a marked increase in the proportion of the protein in the soluble fraction. The missense mutation between CUT2 and HOX shows apparent reduced levels of matrix association. Antibodies against lamin and histone are presented to show that the fractionation has worked. (**d**) The gray-shaded box represents the protein product of an artificial control mutation that has been made in the cDNA of *SATB2* to create a peptide with none of the DNA-binding domains. This is termed the “N-terminal region.” (**e**) Confocal photomicrographs of cells transiently transfected with plasmids containing wild-type and mutant GFP-tagged *SATB2* cDNA under the control of a ubiquitous promoter. The mutation is shown above each photograph. The wild-type, p.Gly515Ser, and p.Gln566Lys cDNAs all produce a granular pattern of fluorescence within the nucleus, the N-terminal regions show a punctate pattern, and the p.Arg389Cys shows a glassy diffuse pattern. (**f, g**) Representative images of cells from independent transient transfections of the wild-type, p.Arg389Cys, p.Gly515Ser, and p.Gln566Lys cDNAs to assess fluorescence loss and recovery after multiple photobleaching using regions of the nucleus (illustrated for this experiment by the red squares on the photomicrographs). This graph shows quantitation of the fluorescence after each photobleach and recovery. The error bars represent standard deviations for the measurements at each time point.

The available crystal structures for CUT1 (1WIZ) and CUT2 (2CSF) were used to predict the effect of the missense variants on the domain structure using the MODELLER program. Each of the mutations was predicted to result in a structural change with alterations in C-terminal boundary of alpha helix 2 and the N-terminal boundary of helix 3 in the affected CUT domain (**Figure 2a**,**b**).

To assess the effect of the missense variants on subcellular localization, we created plasmids expressing wild-type and mutant SATB2 cDNA fused at its N-terminus to a fluorescent marker protein (GFP). Transient transfection of these constructs was performed to determine the effect of the mutations on subcellular localization. Each of the mutant and wild-type fusion proteins was efficiently targeted to the nucleus; however, specific patterns of nuclear localization were apparent. The p.Arg389Cys change gave a more diffuse pattern than the wild-type, p.Gly515Ser, or p.Gln566Lys substitutions (**Figure 2e**). A construct that contained only the region of the SATB2 N-terminal of the DNA-binding domains was targeted to a relatively small number of discrete nuclear foci (**Figure 2d**,**e**). As an indicator of chromatin association dynamics, we used photobleaching of the exogenous fusion proteins within the nuclei of transfected cells. This technique uses a laser to irreversibly bleach the tagged fluorescent protein in a small area within the nucleus and then measures the speed and completeness of the recovery of fluorescence in the bleached area as an indication of the kinetics of protein association with chromatin or nuclear matrix. This indicated that the CUT1 change was associated with the highest level of residual fluorescence after recurrent photobleaching and a recovery rate similar to that of the wild type. Mutations affecting p.Gly515 and p.Gln566 had profiles similar to those with the most prominent loss of fluorescence following recurrent photobleaching and the slowest recovery (**Figure 2f**,**g**).

We then produced tetracyclin-inducible stable HEK293 cell lines for the GFP-tagged wild type and the same missense mutations. This enabled us to use cell fractionation techniques to look for alterations in the distribution of each protein between the soluble, chromatin, and nuclear matrix (**Figure 2c**). This showed a marked increase in the proportion of the p.Arg389Cys protein in the soluble fraction, although chromatin associated protein was still seen. A lower proportion of the total p.Gln566Lys protein was matrix-associated. With this assay, no other obvious differences were apparent.

### Genotype–phenotype correlations

**Figure 3:**
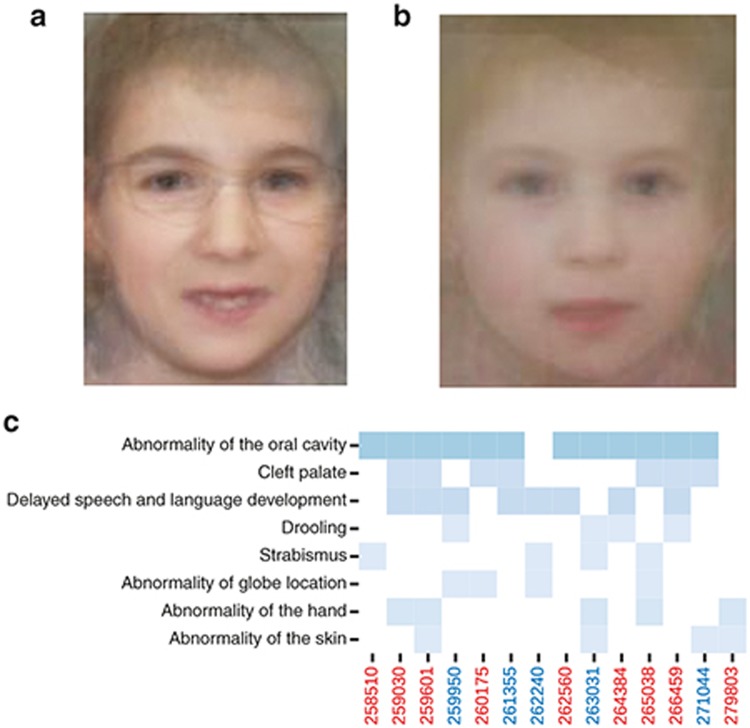
**Facial characteristics associated with de novo mutations in *SATB2*.** (**a**) Average face constructed from the available published facial images from individuals with de novo mutations in *SATB2*. (**b**) Average face constructed from facial photographs from 10 of the individuals with the de novo mutation in *SATB2* reported here. Both images show a small mouth with a thin upper lip and facial asymmetry. The asymmetry is subtle and most noticeable in the jaw line and corners of the mouth. (**c**) Heat map of the recurrently reported human phenotype ontology terms in the 14 Deciphering Developmental Disorders study individuals reported here in whom these terms had been systematically collected prior to the molecular diagnosis being made.

Three approaches were taken to assess the phenotypic characteristics of the individuals with *SATB2* mutations described here. The first compared the facial appearance of published individuals with *SATB2*-associated disease with that of the affected individuals presented here (in whom facial images were available) by creating an “average” face for each group (**Figure 3a**,**b**). This analysis used standard 2D clinical photographs. Both faces were similar, with the main difference being that the individuals reported here appeared younger. The dysmorphism was subtle, with a small mouth and mild facial asymmetry seen in both images. All individuals submitted to DDD are coded using the human phenotype ontology. We used a heat map to display terms that had been used more than once in this group of individuals (**Figure 3c**). No differences were apparent between the individuals in DDD with missense variants or those predicted to have clear loss of function. Finally, aggregation of quantitative and categorical aspects of the phenotype on the basis of the mutation type revealed no significant differences between the missense and loss-of-function groups (**Supplementary Table S1** online).

## Discussion

The individuals reported here were ascertained via diagnostic sequencing approaches that do not require a priori suspicion of any specific clinical syndrome to be available. Since 2010, trio-based next-generation sequencing approaches have transformed our understanding of the generic architecture of moderate to severe global neurodevelopmental disability,^26^ with the proportion of individuals in whom a diagnostic variant can be confidently assigned approaching 40% in previously undiagnosed individuals. The most common genetic—and analytically tractable—mechanism is de novo mutation with protein-altering or haploinsufficient effects. This approach to diagnosis enables a relatively unbiased picture of the phenotype associated with each gene/genetic mechanism to be obtained. *SATB2* is among the most commonly reported genes in the DDD study, accounting for ~0.3% (14/4294) of all analyzed individuals.

For *SATB2*, the core associated clinical features have remained remarkably stable since the original individuals were reported by two of this article’s authors (H.V.F. and D.R.F.) in 1999.^1,2^ These two girls had strikingly similar facial appearances; both had cleft palate, normal prenatal and postnatal growth, and intellectual disability most strikingly in speech and language development. For the individuals reported here, neurodevelopmental impairment was the universal method of ascertainment. Other recurrent clinical features were normal somatic growth in 17/19 affected individuals; 16/19 had absent or near absent speech, with drooling reported for 12/19, suggesting that the development of normal oral motor coordination may be significantly impaired. Close to half of the affected individuals (9/19) had cleft palate. Minor dental anomalies were reported for 8/19. The behavioral phenotype appeared to show the most marked discrepancy within the cohort, with both severe autistic features and friendly/happy personalities being recurrently reported. The overall level of intellectual disability was in the moderate to severe group. Importantly, no differences could be discerned in the range or severity of phenotypes between individuals with clear loss-of-function mutations and those with missense variants, supporting haploinsufficiency as the common pathogenic mechanism.

To objectively assess the facial appearance associated with *SATB2*, we used recently developed image analysis techniques that were previously used for computational assessment of dysmorphic diagnoses.^23,24^ The resulting average face created for the current and published groups suggests that the facial appearance of younger individuals is similar but more subtle than that of the previously reported individuals commonly ascertained via cytogenetic analysis. During this analysis, consistent asymmetry in the face was noted. Asymmetry had previously been noted in both the brain and cranium on high-resolution magnetic resonance imaging as part of a clinical reassessment of one of the original *SATB2* individuals.^16^ Although SATB2 was previously implicated in the coordination of scaled growth between the upper and lower jaw,^27^ it is not known to have a role in maintaining symmetry.

**Figure 4:**
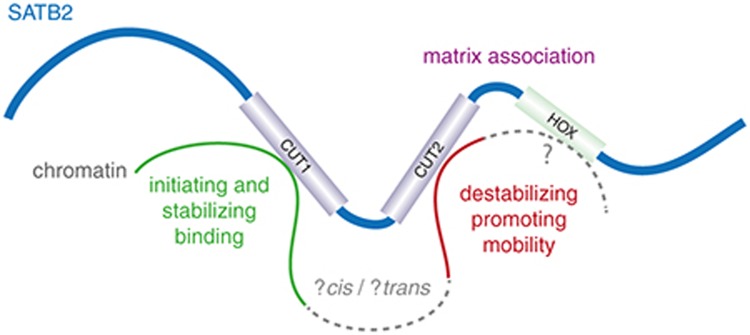
**Provisional model for the role of SATB2 DNA-binding domains.** Cartoon representation of SATB2 interacting with chromatin in vivo. This model shows the binding of CUT1 as an initiating and stabilizing event and CUT2 binding with an antagonistic or destabilizing event that promotes mobility of SATB2 within the nucleus. Uncertainty remains regarding the role of the HOX domain and whether the chromatin that binds to CUT1 and CUT2 is in *cis* or in *trans* with each other. The regions of the protein that mediate matrix attachment are not well defined, but a variant between the CUT2 and HOX domains appears to result in a reduced proportion of the SATB2 being matrix-attached.

Although the precise nuclear function of SATB2 is not yet clear, it binds DNA in a sequence-specific manner in vivo, resulting in gene activation in *cis*^28^ and is expressed in a site-specific and stage-specific manner during development; in light of the human and mouse phenotypes associated with heterozygous loss-of-function mutations, it seems reasonable to consider it to be a dosage-sensitive transcription factor. In addition, it also seems to play a role in establishing higher-order chromatin structure,^28^ suggesting a more fundamental role in complex transcriptional control. There is very little known about how the three DNA-binding domains in SATB2 cooperatively function; however, it is not clear whether they bind DNA in *cis* or in *trans* with each other. For this reason, we were particularly interested in the natural experiments that are represented within the mutations reported here. The missense variants identified within the core of the CUT domain are very likely to result in loss of DNA-binding activity given the predicted effect on the helical structure of the domain. By comparing the effect of mutations in CUT1 and CUT2, we hoped to be able to clarify the role of each. However, the results were surprising. The mutation in CUT1 resulted in a marked increase in mobility of the tagged protein, suggesting that this domain is required to initiate interaction with chromatin. Mutations in CUT2 (and the region between CUT2 and HOX) had the opposite effect, suggesting that this domain is required to facilitate dissociation of SATB2 from bound chromatin. Taking these data together, one possible explanation is that deviation from the normal structure of the C-terminal region results in dramatic changes in the kinetics of chromatin association and that this mimics the complete loss-of-protein function (**Figure 4**).

It is likely that the number of individuals with *SATB2*-associated disorder will increase dramatically over the next few years with the wider use of diagnostic sequencing. In addition to the obvious benefits to our understanding of the clinical consequences of *SATB2* loss of function, the variants themselves should provide useful information that will aid in understanding the basic biology of SATB2 function.

## Disclosure

The authors declare no conflict of interest.
